# The Double Burden of Malnutrition at the Individual Level Among Adults: A Nationwide Survey in the Philippines

**DOI:** 10.3389/fnut.2021.760437

**Published:** 2021-11-15

**Authors:** Aileen R. de Juras, Wan-Chen Hsu, Susan C. Hu

**Affiliations:** ^1^Department of Public Health, College of Medicine, National Cheng Kung University, Tainan City, Taiwan; ^2^Institute of Human Nutrition and Food, College of Human Ecology, University of the Philippines Los Baños, Laguna, Philippines

**Keywords:** double burden of malnutrition, underweight, micronutrient deficiency, overweight, obesity, cardiometabolic risk factors, adults, Philippines

## Abstract

**Introduction:** Double burden of malnutrition (DBM) is a fast-evolving public health challenge. The rising prevalence of obesity and diet-related non-communicable diseases alongside persistent nutritional deficiencies are compelling problems in many developing countries. However, there is limited evidence on the coexistence of these conditions in the same individual among community-dwelling adults. This cross-sectional study describes the various forms of DBM and examines the determinants of DBM at the individual level among adults in the Philippines.

**Materials and Methods:** A nationwide dataset from the 2013 Philippine National Nutrition Survey was used. The final study sample consisted of 17,157 adults (8,596 men and 8,561 non-pregnant and non-lactating women). This study focused on three DBM types within adults: (#1) Underweight and at least one cardiometabolic risk factor (Uw + ≥1 CMRF), (#2) Anemia and at least one cardiometabolic risk factor (An + ≥1 CMRF), (#3) Vitamin A deficiency or iodine insufficiency and at least one cardiometabolic risk factor (Other MND + ≥1 CMRF). The total double burden of malnutrition was also evaluated as the sum of the aforementioned three types. Logistic regression models were used to assess associations between socio-demographic and lifestyle factors and DBM.

**Results:** The prevalence of the three types of DBM were: type #1, 8.1%; type #2, 5.6%; type #3, 20.6%, and the total DBM prevalence was 29.4%. Sex, age, educational attainment, employment status, wealth quintile, and alcohol drinking were the risk factors for DBM. In contrast, marital status, smoking, and physical activity were associated with the different DBM types.

**Conclusion:** The study findings contribute to the current state of knowledge on the broad spectrum of individual-level DBM. Understanding the disparities of this phenomenon could guide integrated actions directed to the concomitance of malnutrition in various forms and cardiometabolic disease risks.

## Introduction

Many developing countries are facing nutrition transitions propelled by socioeconomic and technological advancements (1). These shifts have led to a rise in obesity and non-communicable diseases (NCDs), including hypertension, diabetes mellitus, and cardiovascular diseases (2). Parallel to this, micronutrient deficiency remains highly prevalent in these countries (3). Evidence suggests that nutrition transition contributes to the double burden of malnutrition (DBM) or “the coexistence of undernutrition or micronutrient deficiency along with overweight, obesity, or diet-related NCDs” (4).

The Philippines suffers from this double nutritional burden, as evident in the annual increase of overweight/obesity among adults at 0.73% over 20 years (1993–2013). In addition, the prevalence of cardiometabolic disease risks (overweight/obesity or abdominal obesity, elevated fasting blood glucose, and abnormalities in blood lipid levels) continuously rise. Worse still, chronic energy deficiency, anemia, and vitamin A deficiency are significant public health problems in Filipino adults (5).

Past studies undertaken in the Philippines investigating DBM have primarily focused on the overlap between underweight and overweight/obesity in the population (6–8) or within households (9). Thus, the double burden of malnutrition at the individual level has not been adequately studied. Research documenting the distribution of adults who are underweight or micronutrient deficient and at the same time experiencing cardiometabolic risk factors (CMRF) are also scarce and limited to urban settings (10–15). Hence, our study aims were: (1) to determine the extent of individual-level DBM among Filipino adults, and (2) to identify the association of DBM with socio-demographic and lifestyle characteristics.

## Materials and Methods

### Study Design and Sampling

We analyzed data from the 2013 National Nutrition Survey (NNS) of the Philippines, publicly available at http://enutrition.fnri.dost.gov.ph/site/home.php (16). The NNS is a cross-sectional survey conducted to define the country's food and nutrition situation (5). In brief, the survey adopted the 2003 Master Sample of the National Statistics Office and utilized a stratified three-stage sampling design (17). Data were collected from 35,825 households across 17 regions of the country between June 2013 to April 2014. A complete description of the 2013 NNS survey methodology has been published previously (5, 18).

**Figure 1:**
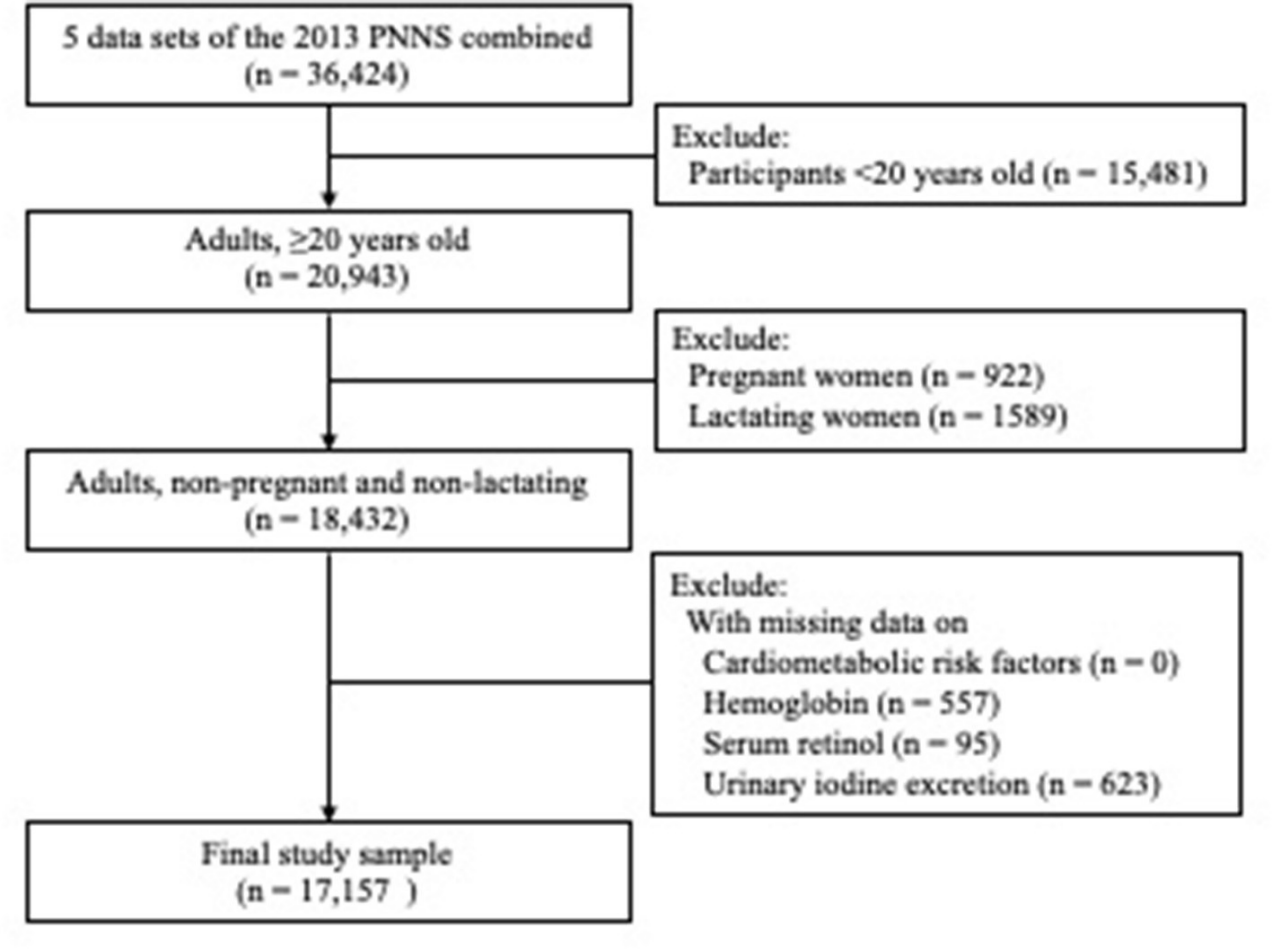
Flow diagram of participants excluded in the study. Cardiometabolic risk factor was defined as an individual with any of the following four factors: (1) overweight/obesity or abdominal obesity; (2) hypertension; (3) hyperglycemia; (4) dyslipidemia (low HDL cholesterol or hypertriacylglycerolemia). There were no study participant with missing value on cardiometabolic risk factor.

This study was limited to adults (≥20 years) with complete subject identification in the anthropometry, biochemical, clinical, and socioeconomic (individual and household) survey components. Moreover, pregnant and lactating women and those with missing values on cardiometabolic risk factors, hemoglobin, serum retinol, and urinary iodine excretion (UIE) were excluded. Accordingly, the final study sample involved 17,157 adults (Figure 1).

### Measurements

#### Anthropometric Data and Cardiometabolic Risk Factors

Weight was measured using the Detecto™ platform beam balance weighing scale to the nearest 0.1 kg, and the Seca™ microtoise was utilized to obtain height to the nearest 0.1 cm (5). We used the weight and height measurements to calculate the body mass index (BMI). The BMI of adults were classified based on the World Health Organization recommended cut-off points: underweight (<18.5 kg/m^2^), normal weight (18.5–24.9 kg/m^2^), overweight (25.0–29.9 kg/m^2^), and obese (≥30.0 kg/m^2^) (19). Waist circumference was obtained with a calibrated tape measure midway between the lowest rib and tip of the hip bone while the participant was standing and breathing normally, and expressed to the nearest 0.1 cm. Abdominal obesity was defined as waist circumference ≥102 cm for males or ≥88 cm for females (20).

Blood pressure readings were taken with a calibrated non-mercurial sphygmomanometer (A&D Um-101™) and stethoscope on the participant's right arm after a minimum of 5 min rest. Systolic and diastolic blood pressures were measured twice with at least 2-min intervals (5). A blood pressure measurement of ≥140/≥90 mmHg indicated hypertension (21).

Venous blood samples were drawn into vacutainers tubes with Lithium Heparin for plasma blood glucose and plain tubes for serum blood lipids. Afterwards, blood glucose and lipid profiles were analyzed utilizing the enzymatic colorimetric method with Roche COBAS Integra and Hitachi 912. The cut-off points for hyperglycemia was fasting blood glucose ≥110 mg/dL (22); low high density lipoprotein (HDL) cholesterol was <40 mg/dL for male or <50 mg/dL for female (23, 24); and hypertriacylglycerolemia was triglyceride ≥150 mg/dL (23, 24). The study participants were then classified as having CMRF if they had any of the following conditions: (1) overweight/obesity or abdominal obesity, (2) hypertension, (3) hyperglycemia, 4) dyslipidemia (low HDL cholesterol or hypertriacylglycerolemia) (14, 25).

#### Micronutrient Deficiency/Insufficiency Indicators

During the survey, hemoglobin, serum retinol, and urinary iodine excretion were collected for the assessment of anemia, vitamin A deficiency, and iodine insufficiency, respectively. Hemoglobin was determined from venous blood samples using the cyanmethemoglobin method (5, 26). Anemia was defined as hemoglobin <13 g/dL for males or <12 g/dL for females (27). Serum retinol was extracted from the blood samples and examined by High-Performance Liquid Chromatography according to the method of Furr et al. (5, 28), where serum retinol of <10 μg/dL indicated vitamin A deficiency (29). Also, midstream urine samples were tested for UIE concentrations. The acid digestion/colorimetric method was employed to evaluate the UIE levels with a cut-off of <50 μg/dL for iodine insufficiency (30, 31).

#### Socio-Demographic and Lifestyle Characteristics

The covariates in this study included sex, age, educational attainment, marital status, employment status, household size, wealth quintile, smoking, alcohol drinking, and physical activity. The participants' sex was classified as male or female. The age variable was categorized as 20–39, 40–59, and ≥60 years old, while educational attainment was grouped into elementary or lower, high school, and college or higher. Marital status was reported as single, married or with a partner, and others (widowed/separated/annulled/divorced). The status of employment utilized binary categories of employed and unemployed. Household size was constructed from the socioeconomic data sets and divided into three levels (1–3, 4–6, and ≥7). Wealth status, based on quintiles of household assets, was created utilizing principal component analysis (5). An individual who smoked either: (1) ≥1 cigarette daily/on a regular or occasional basis, or (2) not daily, but at least weekly/less often than weekly was considered as a current smoker (32). Current alcohol drinking was characterized as consumption of any alcoholic beverage during the past year (33). Low physical activity referred to not having: (1) ≥3 days of vigorous-intensity activity for at least 20 min per day, or (2) ≥5 days of moderate-intensity activity for at least 30 min per day (32).

### Types of Double Burden of Malnutrition

We assessed three types of DBM at the individual level and each was a combination of nutritional deficiency along with any CMRF. Nutritional deficiency encompasses underweight and micronutrient deficiency/insufficiency. CMRF measurements included the following: overweight/obesity or abdominal obesity, hypertension, hyperglycemia, and dyslipidemia (low HDL cholesterol or hypertriacylglycerolemia) (14, 25). Thus, we categorized the three types of DBM as: (#1) co-occurrence of underweight and at least one CMRF (Uw + ≥1 CMRF), (#2) co-occurrence of anemia and at least one CMRF (An + ≥1 CMRF), (#3) co-occurrence of vitamin A deficiency or iodine insufficiency and at least one CMRF (Other MND + ≥1 CMRF). The total double burden of malnutrition was also reported as the sum of the three types (i.e., co-occurrence of underweight or anemia or vitamin A deficiency or iodine insufficiency and at least one CMRF). Since the prevalence of vitamin A deficiency was too small, we did not consider it as a different type.

### Statistical Analysis

All analyses were performed using R software version 4.0.3 (R Foundation for Statistical Computing, Vienna, Austria), and survey sampling weights were employed to obtain results representative of adult population in the Philippines. We generated descriptive statistics for the socio-demographic and lifestyle characteristics of the participants. The prevalence of underweight, overweight, obesity, cardiometabolic risk factors, micronutrient deficiency/insufficiency, and types of DBM were calculated. The chi-square test was utilized to identify the differences between sex and independent variables. We also conducted a bivariate analysis to describe variations with every determinant in each type of DBM and total DBM. Binary logistic regression was carried out to examine the relationships between DBM and socio-demographic and lifestyle factors. Multi-collinearity was assessed in all models, which were all <3, indicating no collinearity. The statistical significance of associations was identified at *p* < 0.05.

## Results

### Characteristics of Study Participants

A total of 17,157 adults were included in the present study, with a balance between male and female participants (Table 1). The study samples were mostly in the 20–39 years age group (45.8%), finished high school education (38.2%), married or with a partner (67.0%), and employed (60.0%). There were slightly more females in the older age group (17.1%) and similarly, more females graduated from college or higher (31.8%). A higher proportion of males (26.5%) were single or unmarried, and 57.1% of females were unemployed.

**Table 1:** Socio-demographic and lifestyle characteristics of study participants^a^.

**Characteristics^b^**	**Total**	**Male**	**Female**	***p-*value**
	**(*n* = 17,157)**	**(*n* = 8,596)**	**(*n* = 8,561)**	
Age group				<0.001
20–39 years	45.8	48.6	43.0	
40–59 years	38.7	37.4	39.9	
≥60 years	15.5	14.0	17.1	
Educational attainment				0.001
Elementary and lower	32.1	33.9	30.3	
High school	38.2	38.4	38.0	
College and higher	29.7	27.7	31.8	
Marital status				<0.001
Single	23.2	26.5	20.0	
Married	67.0	68.3	65.7	
Others	9.8	5.3	14.3	
Employment status				<0.001
Employed	60.0	76.9	42.9	
Unemployed	40.0	23.1	57.1	
Household size				0.289
1–3	33.2	32.9	33.4	
4–6	44.5	44.3	44.7	
≥7	22.3	22.8	21.9	
Wealth quintile				<0.001
Poorest	17.4	19.1	15.6	
Poor	19.3	20.3	18.3	
Middle	20.6	20.7	20.5	
Rich	21.2	20.3	22.1	
Richest	21.5	19.6	23.5	
Current smoker				<0.001
Yes	27.1	46.1	8.2	
No	72.9	53.9	91.8	
Current alcohol drinker				<0.001
Yes	51.5	72.7	30.4	
No	48.5	27.3	69.6	
Physical activity				<0.001
Low	43.5	35.6	51.4	
High	56.5	64.4	48.6	

a *Values are weighted percentages (%)*.

b *Variables with missing observations: educational attainment (n = 72), smoking and drinking status (n = 988), physical activity classification (n = 1,176)*.

Approximately 45% of the study participants belonged to households with 4–6 members, and no differences in sex were found. Regarding wealth status, more males were in the poorest and poor quintiles. For lifestyle factors, 27.1% were current smokers, 51.5% were current alcohol drinkers, and 43.5% had low physical activity. A markedly greater percentage of males were smokers and drinkers at the time of the survey. Inversely, more females had low physical activity.

### Nutritional Status of Study Participants

The prevalence of underweight, overweight, and obesity was 11.2, 23.4, 6.2%, respectively, and these conditions were consistently higher in females than males (Table 2). A large proportion of the participants (84.4%) had at least one CMRF, which was higher in females. Among the metabolic syndrome components, the highest prevalence was for low HDL cholesterol (70.4%), while the lowest was hyperglycemia (9.9%). These components, except for hyperglycemia, showed statistical differences between males and females. Remarkably, abdominal obesity prevalence among females was seven times higher than males (20.8 vs. 2.9%). As for micronutrient deficiency/insufficiency, iodine insufficiency had the greatest prevalence (23.9%), whereas vitamin A deficiency was noted in a very small percentage of adults (0.1%). More female adults were affected by anemia and iodine insufficiency.

**Table 2:** Nutritional status, cardiometabolic risk factors, and double burden of malnutrition of adults in the Philippines^a^.

**Variables**	**Total**	**Male**	**Female**	***p-*value**
	**(*n* = 17,157)**	**(*n* = 8,596)**	**(*n* = 8,561)**	
Body mass index				<0.001
Underweight	11.2	10.6	11.8	
Normal	59.3	63.2	55.3	
Overweight	23.4	21.5	25.3	
Obese	6.2	4.6	7.7	
Metabolic syndrome components				
Abdominal obesity	11.8	2.9	20.8	<0.001
Hypertension	22.7	24.5	20.9	0.003
Hyperglycemia	9.9	10.4	9.3	0.056
Low HDL cholesterol	70.4	61.9	78.8	<0.001
Hypertriacylglycerolemia	39.5	46.6	32.4	<0.001
≥1 Cardiometabolic risk factor^b^, ^c^	84.4	81.6	87.2	<0.001
Micronutrient deficiency/insufficiency				
Anemia	6.5	5.3	7.8	0.002
Vitamin A deficiency	0.1	0.1	0.1	0.474
Iodine insufficiency	23.9	21.5	26.2	0.001
≥1 MND	28.5	25.4	31.6	<0.001
Double burden of malnutrition^d^				
#1. Uw + ≥1 CMRF	8.1	7.2	8.9	0.011
#2. An + ≥1 CMRF	5.6	4.3	6.9	0.001
#3. Other MND + ≥1 CMRF	20.6	17.9	23.2	<0.001
Sum of the three types (Total DBM)	29.4	25.7	33.1	<0.001

a *Values are weighted percentages (%)*.

b *Cardiometabolic risk factor was defined as an individual with any of the following four factors: (1) overweight/obesity or abdominal obesity; (2) hypertension; (3) hyperglycemia; (4) dyslipidemia (low HDL cholesterol or hypertriacylglycerolemia)*.

c *Cardiometabolic risk factor with missing observations: both body mass index and abdominal obesity (n = 370), hypertension (n = 66), hyperglycemia (n = 651), both low HDL cholesterol and hypertriacylglycerolemia (n = 206)*.

d *#1. Uw + ≥1 CMRF, co-occurrence of underweight and at least one cardiometabolic risk factor; #2. An + ≥1 CMRF, co-occurrence of anemia and at least one cardiometabolic risk factor; #3. Other MND + ≥1 CMRF, co-occurrence of vitamin A deficiency or iodine insufficiency and at least one cardiometabolic risk factor; Sum of the three types or Total DBM. HDL, High-density lipoprotein; Uw, Underweight; CMRF, Cardiometabolic risk factor; An, Anemia; MND, Micronutrient deficiency/insufficiency; DBM, double burden of malnutrition*.

Overall, 29.4% adults exhibited a double burden of malnutrition and was significantly higher for females than males. The prevalence of the three types of DBM was: (#1) the co-occurrence of underweight and at least one CMRF was 8.1%; (#2) the co-occurrence of anemia and at least one CMRF was 5.6%; and (#3) the co-occurrence of vitamin A deficiency or iodine insufficiency and at least one CMRF was 20.6%.

### Bivariate Associations Between Socio-Demographic and Lifestyle Factors and DBM

As shown in Table 3, significant differences between socio-demographic and lifestyle factors and DBM were identified. The prevalence of DBM was higher among females, aged ≥60 years old, with elementary education or below, widowed/separated/annulled/divorced, unemployed, residing in small-sized (1–3 members) and poorest households, and non-drinkers. To add, current smoking was correlated with the three DBM types. Significant differences were also observed in DBM types #2 and #3 for physical activity.

**Table 3:** Socio-demographic and lifestyle characteristics of adults by double burden of malnutrition characterizations^a^.

**Variables^b^**		**Type #1^c^**		**Type #2^c^**		**Type #3^c^**		**Total DBM^c^**	
		**(*n* = 1,524)**	***p-*value**	**(*n* = 1,066)**	***p-*value**	**(*n* = 3,946)**	***p-*value**	**(*n* = 5,529)**	***p-*value**
Sex	Male	7.2	0.011	4.3	0.001	17.9	<0.001	25.7	<0.001
	Female	8.9		6.9		23.2		33.1	
Age group	20–39 years	7.3	<0.001	3.3	<0.001	15.1	<0.001	23.1	<0.001
	40–59 years	6.2		5.7		23.3		31.0	
	≥60 years	15.3		11.9		29.8		44.4	
Educational attainment	Elementary and lower	12.0	<0.001	7.9	<0.001	26.1	<0.001	37.6	<0.001
	High school	7.4		5.1		19.2		27.9	
	College and higher	4.6		3.7		16.3		22.4	
Marital status	Single	9.5	<0.001	3.1	<0.001	15.4	<0.001	24.5	<0.001
	Married	6.6		5.7		21.2		29.3	
	Others	14.5		11.1		28.1		42.0	
Employment status	Employed	6.4	<0.001	4.3	<0.001	19.1	0.003	26.4	<0.001
	Unemployed	10.7		7.6		22.7		34.0	
Household size	1–3	8.8	0.053	6.7	0.006	22.3	0.026	32.0	0.005
	4–6	7.3		5.1		19.7		28.1	
	≥7	8.4		5.0		19.6		28.3	
Wealth quintile	Poorest	13.9	<0.001	6.5	0.014	26.1	<0.001	38.5	<0.001
	Poor	10.0		6.2		24.1		34.3	
	Middle	8.3		6.2		21.2		30.4	
	Rich	5.7		5.1		17.7		24.9	
	Richest	3.9		4.4		15.1		21.3	
Current smoker	Yes	9.9	0.005	4.6	0.026	19.0	0.032	28.3	0.097
	No	7.5		6.0		21.3		30.1	
Current alcohol drinker	Yes	6.7	0.002	3.9	<0.001	17.6	<0.001	25.0	<0.001
	No	9.6		7.4		24.0		34.5	
Physical activity	Low	8.8	0.058	6.3	0.043	19.6	0.036	29.4	0.549
	High	7.6		5.2		21.7		29.9	

a *Values are weighted percentages (%)*.

b *Variables with missing observations in Type #1: educational attainment (n = 11), smoking and drinking status (n = 82), physical activity classification (n = 98); in Type #2: educational attainment (n = 11), smoking and drinking status (n = 54), physical activity classification (n = 64); in Type #3: educational attainment (n = 19), smoking and drinking status (n = 202), physical activity classification (n = 235); in Total DBM: educational attainment (n = 31), smoking and drinking status (n = 286), physical activity classification (n = 337)*.

c *Type #1. Uw + ≥1 CMRF, co-occurrence of underweight and at least one cardiometabolic risk factor; Type #2. An + ≥1 CMRF, co-occurrence of anemia and at least one cardiometabolic risk factor; Type #3. Other MND + ≥1 CMRF, co-occurrence of vitamin A deficiency or iodine insufficiency and at least one cardiometabolic risk factor; Total DBM, Total double burden of malnutrition or sum of the three types*.

### Determinants of the Double Burden of Malnutrition

Table 4 displays the socio-demographic characteristics, lifestyle factors, and types of DBM in the logistic regression models. We found that sex, age, educational attainment, employment status, wealth quintile, and alcohol drinking were associated with DBM at the individual level. Being a female had higher odds of experiencing DBM. Older adults (≥60 years) were twice likely to have DBM compared with younger adults (20–39 years). Furthermore, middle-aged adults (40–59 years) had a lower risk of DBM type #1 but not for the other types. Adults with higher education, unemployed, and non-current drinkers were significantly associated with DBM. Noteworthy, the odds of having DBM declined with improvement in the wealth quintile.

**Table 4:** Factors associated with the double burden of malnutrition characterizations^a^.

**Variables**	**Type #1^b^**	**Type #2^b^**	**Type #3^b^**	**Total DBM^b^**
	**(*n* = 1,524)**	**(*n* = 1,066)**	**(*n* = 3,946)**	**(*n* = 5,529)**
Female (ref = male)	**1.21 (1.03, 1.42)**	1.16 (0.97, 1.39)	**1.24 (1.13, 1.37)**	**1.26 (1.14, 1.39)**
Age group (ref = 20–39)				
40–59	**0.98 (0.82, 1.17)**	**1.58 (1.29, 1.95)**	**1.59 (1.42, 1.77)**	**1.47 (1.33, 1.63)**
≥60	**2.13 (1.72, 2.64)**	**2.63 (2.10, 3.30)**	**2.04 (1.78, 2.34)**	**2.27 (2.00, 2.57)**
Educational attainment				
Elementary and lower (ref)				
High school	**0.85 (0.73, 0.98)**	0.89 (0.75, 1.06)	0.90 (0.81, 1.01)	**0.89 (0.81, 0.98)**
College and higher	**0.63 (0.51, 0.78)**	**0.77 (0.62, 0.95)**	0.92 (0.80, 1.05)	**0.83 (0.74, 0.94)**
Marital status (ref = single)				
Married	**0.52 (0.44, 0.63**)	**1.33 (1.04, 1.71)**	1.05 (0.92, 1.19)	0.91 (0.81, 1.02)
Others	0.79 (0.62, 1.02)	**1.66 (1.24, 2.23)**	1.13 (0.96, 1.34)	1.09 (0.93, 1.28)
Unemployed (ref = employed)	**1.57 (1.36, 1.82)**	**1.51 (1.28, 1.79)**	**1.13 (1.03, 1.24)**	**1.27 (1.16, 1.38)**
Household size (ref = 1–3)				
4–6	1.03 (0.89, 1.20)	0.90 (0.77, 1.04)	0.96 (0.87, 1.06)	0.97 (0.89, 1.05)
≥7	1.17 (0.97, 1.41)	0.88 (0.71, 1.10)	1.03 (0.90, 1.17)	1.03 (0.92, 1.16)
Wealth quintile (ref = poorest)				
Poor	**0.74 (0.62, 0.87)**	0.92 (0.73, 1.16)	0.95 (0.82, 1.10)	**0.88 (0.78, 0.99)**
Middle	**0.59 (0.49, 0.72)**	0.95 (0.76, 1.20)	**0.81 (0.69, 0.93)**	**0.73 (0.64, 0.83)**
Rich	**0.39 (0.32, 0.49)**	0.81 (0.63, 1.04)	**0.65 (0.55, 0.77)**	**0.56 (0.49, 0.64)**
Richest	**0.30 (0.23, 0.40)**	**0.73 (0.56, 0.96)**	**0.54 (0.45, 0.65)**	**0.47 (0.41, 0.55)**
Non-smoker (ref = yes)	**0.62 (0.53, 0.72)**	1.00 (0.82, 1.21)	1.01 (0.91, 1.13)	0.92 (0.83, 1.01)
Non-alcohol drinker (ref = yes)	**1.28 (1.10, 1.50)**	**1.38 (1.16, 1.63)**	**1.20 (1.09, 1.33)**	**1.27 (1.16, 1.39)**
High physical activity (ref = low)	0.93 (0.82, 1.06)	0.94 (0.80, 1.10)	**1.18 (1.07, 1.30)**	1.08 (0.99, 1.18)

*The values in bold are significantly different at a level of p < 0.05*.

a *Values are odds ratios (ORs) and 95% confidence intervals (CIs). All models were controlled for variables shown in the first column*.

b *Type #1. Uw + ≥1 CMRF, co-occurrence of underweight and at least one cardiometabolic risk factor; Type #2. An + ≥1 CMRF, co-occurrence of anemia and at least one cardiometabolic risk factor; Type #3. Other MND + ≥1 CMRF, co-occurrence of vitamin A deficiency or iodine insufficiency and at least one cardiometabolic risk factor; Total DBM, Total double burden of malnutrition or sum of the three types*.

The variables of smoking and physical activity were associated only with a single type of DBM. Not smoking offered protection for DBM type #1, while engaging in high physical activity increased DBM type #3 risk. For marital status, those who were married had lower susceptibility to DBM type #1. No significant differences were noted for household size in any type of DBM.

## Discussion

To the best of our knowledge, this current study is one of the first to assess the broad spectrum of DBM at the individual level in Southeast Asia. The results highlight that nutritional deficiencies (underweight or micronutrient deficiency/insufficiency) simultaneously occur with cardiometabolic risk factors in the adult population. Moreover, the distribution of DBM varies across several socio-demographic and lifestyle parameters.

The prevalence of underweight, micronutrient deficiency/insufficiency, and cardiometabolic risk factors in this study were comparable with national estimates and past literature (34–36). Notably, overweight was twice higher than underweight in the adult population. For the dual burden of malnutrition, the overall DBM prevalence and DBM type #1 were higher than published studies (14, 15). However, caution must be taken when comparing the extent of DBM given the differences in the stage of nutrition transition across different countries, i.e., the Philippines is in the more advanced phase, whereas Burkina Faso is in the early phase (37, 38).

A wide range of socio-demographic and lifestyle factors was associated with DBM. Women bear an enormous burden of DBM, and late adulthood increases the odds of experiencing DBM. Females and older adults in the Philippines are at greater independent risk of having undernutrition, micronutrient deficiencies, and cardiometabolic disease risks (5). Likewise, the strong DBM determinant of age may be ascribed to the biological and environmental changes in aging that may contribute to the development of DBM (4). This finding suggests that DBM may progressively become an age-related disease, and must be addressed as life expectancy continues to increase in the future.

Educational attainment, marital status, employment, and wealth quintile were the other DBM risk factors. A higher educational level was found to reduce the risk of DBM possibly through better dietary and lifestyle choices (39). Being married and widowed/separated/annulled/divorced was also related to DBM. These life transitions are stressful events that could positively or negatively alter an individual's psychosocial condition affecting their nutritional status. It was also hypothesized that the availability of financial resources and social support systems could also influence marital and health states (40, 41). Adults without employment were more vulnerable to any type of DBM. This is in line with a past research wherein underweight and anemia prevalence was highest among unemployed adults (35). In the same study, adults with no employment were less physically active than those who were employed, which may further explain this relationship (35). The association of household wealth with DBM indicates that these conditions are not a matter of affluence, i.e., it could happen in different wealth statuses. Angeles-Agdeppa et al. documented that an advancement in wealth among Filipino households was not always translated to better dietary intakes (42).

Smoking, alcohol drinking, and physical activity were the lifestyle factors correlated to DBM. Adults who were not currently smoking had lesser susceptibility to DBM type #1. Existing literature on tobacco use suggests that smoking increases the risk for non-communicable diseases through weight gain, inflammatory reactions, and oxidative stress (43, 44). Conversely, non-current drinkers and those physically active had higher a risk for DBM. It should be noted that non-current drinkers in this study covered adults who were lifetime abstainers or former drinkers. Alcohol consumption could affect nutrient metabolism and absorption, which may then lead to malnutrition. Evidence substantiates the adverse effects of alcohol intake on nutritional deficiencies and cardiometabolic disease risks (45, 46). It is widely known that exercise with optimal duration and intensity decreases the risk of cardiovascular disease. Nonetheless, individuals who engage in physical activity may undergo modest and short-term changes in cardiometabolic disease risks (47–49). Taken altogether, these DBM determinants are potentially modifiable, and underlines that an integrated approach is vital to prevent malnutrition in all its forms.

The main strength of this study is the use of a large nationally representative dataset with biochemical markers for the investigation of micronutrient deficiencies and cardiometabolic risk factors. Nevertheless, we must interpret the study findings while considering some limitations. First, the cross-sectional study design did not allow for the life-course analysis of DBM, and the observed associations to be interpreted as causal. Second, unmeasured non-nutritional factors, i.e., inflammation, medication use, and disease history, may have biased the study. Third, we did not include data on food consumption that could help explain the nutritional outcomes. Finally, bias due to the missing variable may be limited because the models were adjusted for potentially important determinants of DBM that were not collinear.

To conclude, our study findings confirm the persistence of undernutrition amidst cardiometabolic risk factors among Filipino adults. The overall prevalence of individual-level DBM was 29.4%. Sex, age, educational attainment, employment status, wealth quintile, and alcohol drinking were the factors related to DBM. On the other hand, marital status, smoking, and physical activity were associated with the different types of DBM. These factors alter the various forms of malnutrition and cardiometabolic risk factors through unhealthy diets and lifestyles. Given the ongoing nutrition transition in the Philippines, it is imperative to tackle these nutritional problems simultaneously through double-duty actions. This may involve programs focusing on women's nutrition, and social policies such as continued investments in education and employment opportunities. A holistic and intensified approach to promote healthy diets and lifestyles is equally important to improve the nutritional landscape of adults.

## Data Availability Statement

Publicly available datasets were analyzed in this study. This data can be found here: http://enutrition.fnri.dost.gov.ph/site/home.php.

## Ethics Statement

The studies involving human participants were reviewed and approved by Human Research Ethics Committee of National Cheng Kung University, Tainan City, Taiwan. The patients/participants provided their written informed consent to participate in this study.

## Author Contributions

AJ, W-CH, and SH designed the research. AJ wrote the first draft of the manuscript. W-CH and AJ analyzed the data. SH supervised the research and had primary responsibility for the final content. All authors have read and approved the submitted version of the manuscript.

## Conflict of Interest

The authors declare that the research was conducted in the absence of any commercial or financial relationships that could be construed as a potential conflict of interest.

## Publisher's Note

All claims expressed in this article are solely those of the authors and do not necessarily represent those of their affiliated organizations, or those of the publisher, the editors and the reviewers. Any product that may be evaluated in this article, or claim that may be made by its manufacturer, is not guaranteed or endorsed by the publisher.
